# PRAF2 overexpression predicts poor prognosis and promotes tumorigenesis in esophageal squamous cell carcinoma

**DOI:** 10.1186/s12885-019-5818-7

**Published:** 2019-06-14

**Authors:** Zhaoye Qian, Bin Wei, Yu Zhou, Qiuzi Wang, Jiru Wang, Yuan Sun, Yong Gao, Xiaofei Chen

**Affiliations:** Department of Medical Oncology, The Affiliated Huaian No.1 People’s Hospital of Nanjing Medical University, Huai’an, 223300 China

**Keywords:** PRAF2, Prognosis, Proliferation, Invasion, Apoptosis, Esophageal squamous cell carcinoma

## Abstract

**Background:**

Prenylated Rab acceptor 1 domain family, member 2 (PRAF2) is involved in the occurrence and progression of several malignant tumors. However, its potential role in esophageal squamous cell carcinoma (ESCC) is still unknown.

**Methods:**

PRAF2 mRNA expression was determined in 77 frozen ESCC samples by quantitative reverse transcription-polymerase chain reaction (qPCR) and its association with clinical features and overall survival were evaluated. The roles of PRAF2 in ESCC cells were investigated by proliferation, cell cycle, invasion and apoptosis assays in vitro.

**Results:**

The PRAF2 mRNA expression was significantly increased in ESCC tissues compared with matched surrounding non-tumor tissues. Survival analysis showed that high PRAF2 mRNA expression was associated with worse overall survival in ESCC patients. Multivariate analysis revealed that PRAF2 (hazard ratio 2.05, 95% CI 1.10–3.85, *P* = 0.025) emerged as the independent predictor for poor overall survival in ESCC. The in vitro experiments revealed that knockdown of PRAF2 expression blocked cell proliferation, cell cycle progression and cell invasion and induced cell apoptosis in ESCC cells.

**Conclusion:**

Taken together, our data demonstrate that PRAF2 could be used as a potential prognostic biomarker and represent a potential therapeutic target for ESCC.

## Background

Esophageal cancer is the eighth most common cancer with an increasing morbidity and mortality rates worldwide [1, 2]. About 90% of esophageal cancers are esophageal squamous cell carcinoma (ESCC) on pathological examination [3]. Despite the progress in diagnosis and treatment, the overall five-year survival rate of ESCC is 15 to 25% due to diagnosis in later stages [4, 5]. Therefore further elucidation of the molecular events in ESCC development may yield alternative means for the management of ESCC [6, 7].

Prenylated Rab acceptor 1 domain family, member 2 (PRAF2, also called JM4), a 178 amino acid endoplasmic reticulum (ER)-resident protein, comprise a prenylated Rab acceptor motif and four transmembrane domains [8, 9]. PRAF2, which is strongly expressed in human normal tissues [10], has been identified as a novel protein involved in ER-to-Golgi transport and vesicular traffic [11–13]. Unlike the PRAF1 and PRAF3 (also belong to the PRA family) well characterized in tumorigenesis [14–17], PRAF2 has not been investigated until quite recently. Now, PRAF2 has been considered as oncogene since it is highly expressed in multiple tumor tissues of the breast, colon, lung and ovary cancer [10]. It has been reported that overexpression of PRAF2 shows a significantly correlation with malignant clinical characteristics and predicts poor prognostic outcome in malignant glioma, neuroblastoma and hepatocellular carcinoma [18–20]. Moreover, PRAF2 may play important roles in the malignant transformation of above tumor types [18–21]. In molecular mechanism, monomeric/dimeric state of PRAF2 may influence its subcellular localization and distribution that leads to functional differences in malignant glioma cells [18]. Conversely, Vento et al. reported that PRAF2 may exert as a tumor suppressor gene for its function in inducing apoptotic cell death through interacting with Bcl-xL/Bcl-2 and translocating Bax to mitochondria [22]. However, the expression profile and biological role of PRAF2 in ESCC have not been explored.

In present study, we find that PRAF2 expression was an independent prognostic biomarker for poor survival in patients with ESCC. Furthermore, knockdown of PRAF2 expression in ESCC cells blocked cell growth and migration and stimulated cell apoptosis.

## Methods

### Patients and sample collection

A total of 77 ESCC patients and relevant clinical data (including age, gender, tumor location, clinical stage, and differentiation grade) were obtained from The Affiliated Huaian No.1 People’s Hospital of Nanjing Medical University (Huai’an, Jiangsu Province, China) from May 2010 to June 2014. Overall survival (OS) was calculated from the date of diagnosis to the date of last follow-up or mortality from any cause. The surgically resected tumor tissues and matched surrounding non-tumor tissues were obtained from patients for further analysis of gene expression. Written informed consent for the use of tissue specimens was obtained from all patients, and the protocol for the present study was approved by the Ethics Committee of The Affiliated Huaian No.1 People’s Hospital of Nanjing Medical University.

### Cell culture and transfection

The TE1 cell line (no. TCHu 89; cell line was last tested and authenticated on January 8, 2018 by STR Profiling) was obtained from the Cell Bank of the Chinese Academy of Sciences (Shanghai, China) in 2016. Cells were cultured in DMEM media (KeyGEN, Nanjing, China) supplemented with 10% fetal bovine serum (Gibco, Grand Island, USA), and cultured at 37 °C in a humidified incubator containing 5% CO^2^. PRAF2 silencing was achieved using small interfering RNA (siRNA). Transfection of siRNA was performed using Lipofectamine 2000 Reagent (Invitrogen, Carlsbad, USA). The PRAF2 siRNA (siPRAF2) sequence was 5′-UCAACAACCUCCUCUACUA-3′ and the negative control sequence was 5′-UAGUAGAGGAGGUUGUUGA-3′ (RiboBio, Guangzhou, China).

### Cell proliferation analysis

For the Cell Counting Kit-8 (CCK-8; Dojindo, Japan) assay, transfected cells were plated in a 96-well plate at a density of 2000 cells/100 μL and cultured for 24, 48, and 72 h, respectively. Then the absorbance was measured at 450 nm using a microplate reader (BioTek, Winooski, USA). For colony formation assay, a total of 200 cells were placed in a fresh 6-well plate and cultured in medium containing 10% fetal bovine serum, with medium replacement every 3–4 days. After 2 weeks, cells were fixed with 4% paraformaldehyde and stained with 0.1% crystal violet. Visible colonies were manually counted.

### Cell cycle analysis

After transfection, the TE1 cells were collected and subjected to PI staining using the CycleTEST™ PLUS DNA Reagent Kit (BD Biosciences, Franklin Lakes, USA). Next, Flow cytometry (BD Biosciences, Franklin Lakes, USA) analysis was performed to detect cell cycle distribution. Finally, the percentage of cells in G1, S, and G2-M phases were analyzed by the ModFit program.

### Invasion assays

Cell invasion were examined using transwell assay. Briefly, transfected cells were resuspended in 100 μl serum-free medium and added to the upper chamber of transwell filter, containing a Matrigel-coated membrane (BD Biosciences, Franklin Lakes, USA). The lower chamber was filled with 500 μl medium containing 20% fetal bovine serum. After incubation, cells that migrated to the lower compartment were fixed with methanol, stained with crystal violet in each well. Imaging and cell counting were performed at × 10 objective lens under a light microscope.

### Apoptotic analysis

The Annexin V-FITC/PI Apoptosis Detection Kit (BD Biosciences, Franklin Lakes, USA) was used to detect apoptotic cells. Cells were collected after transfection and mixed with Annexin V-FITC/PI buffer. Flow cytometry (BD Biosciences, San Diego, USA) was used to identify cells of normal status, early apoptosis, late apoptosis and death. The relative ratios of cells in the initial stages of apoptosis were analyzed.

### Quantitative real-time polymerase chain reaction (qPCR)

After frozen specimens (~ 10 mg/per) were ground in liquid nitrogen and TE1 cells collected, the total RNA was isolated with TRIzol reagent (Invitrogen, Carlsbad, USA) and reverse with transcripted with FastQuant RT Kit (Tiangen, Beijing, China). Then, PCR reaction was performed in triplicate with SuperReal PreMix Plus (Tiangen, Beijing, China) using the Real-Time PCR Detection System (Roche, California, USA). The specific primers for PRAF2 were forward: 5′ -GCCGGTGCTTCTGATCCTG- 3′ and reverse: 5′ -GATCCAGCCTCCTGCTCTTG- 3′. The forward and reverse primers for GAPDH were 5′ -ACCAGCCTCAAGATCATCAGC- 3′ and 5′ -TGCTAAGCAGTTGGTGGTGC- 3′, respectively. The qPCR results were analyzed and expressed as relative expression of threshold cycle (Ct) value, which was then converted to fold changes.

### Western blot

Transfected cells were harvested and treated with RIPA lysis buffer (KeyGEN, Nanjing, China) on ice, and protein concentration was determined using a BCA Kit (KeyGEN, Nanjing, China). Equal amounts of total protein were loaded on SDS-PAGE gels and subjected to electrophoresis. After separation on the gel, proteins were transferred to a PVDF membrane. The primary antibodies against PRAF2, BCL-XL, BAX, caspase3, cleaved-caspase3, caspase9, cleaved-caspase9 and GAPDH (Abcam, Cambridge, UK) were used according to the manufacturers’ recommendations. Then membranes were incubated with HRP-conjugated secondary antibody (Abcam, Cambridge, UK) and visualized by using ECL detection (KeyGEN, Nanjing, China).

### TCGA verification of hub genes

UALCAN (http://ualcan.path.uab.edu) was applied to construct an algorithm on the basis of The Cancer Genome Atlas (TCGA) database [23]. The expression level of PRAF2 was verified in UALCAN esophageal cancer database.

### Statistical analysis

All results are presented as the mean ± SD. Pearson’s chi-squared test, Fisher’s exact test, Kaplan-Meier method (log-rank test), multivariate Cox regression analysis, Student’s t-test and Kruskal-Wallis test were used to analyze the data using SPSS Statistics software (version 19.0, Chicago, USA). Relative PRAF2 expression in each tumor was calculated by normalizing to matched normal tissue, and the mean fold-change was employed as the cut-off points for case grouping. All cell experiments were performed in triplicate. *P* < 0.05 was considered statistically significant. Graphs were made using the GraphPad Prism 6.0 software package (La Jolla, CA, USA).

## Results

### PRAF2 is highly expressed in ESCC tissues, and correlates with clinical characteristics

**Fig. 1:**
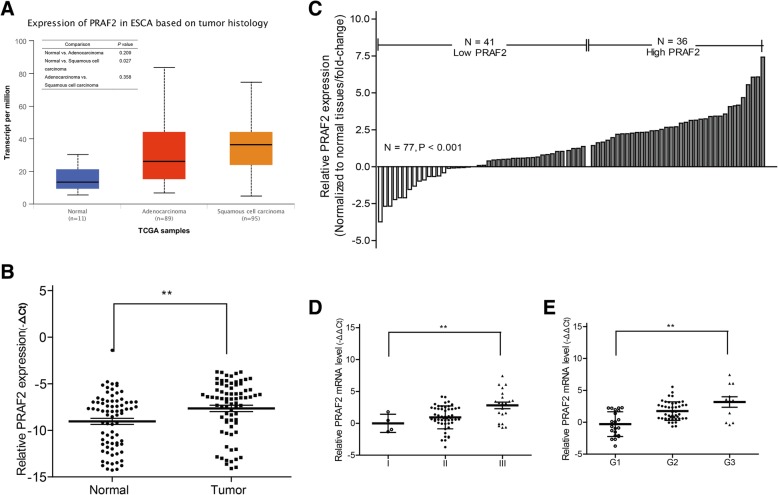
PRAF2 is highly expressed in ESCC tissues. **a** Expression of PRAF2 in ESCC tissues compared to normal tissues using TCGA samples. **b** qPCR analysis of PRAF2 mRNA expression in 77 paired ESCC tissues and their adjacent non-tumor tissues. PRAF2 levels were normalized to GAPDH expression. **c** Patients with ESCC were divided into two groups according to their PRAF2 expression profiles. **e**, **f** Correlation of PRAF2 mRNA expression with differentiation grade and TNM stage. ** *P* < 0.01

In present study, we first used TCGA data of ESCC patients via the UALCAN data portal and found that the PRAF2 were up-expressed in squamous cell carcinoma but not in adenocarcinoma tissues compared to normal tissues (Fig. 1a, *P* = 0.027). Then, we collected the cancer and matched associated non-tumor from ESCC patients and tested the PRAF2 mRNA expression by qPCR method. PRAF2 mRNA expression was markedly elevated in cancer tissues compared with non-cancerous normal tissues (Fig. 1b). To further explore the correlation between PRAF2 mRNA expression and clinical characteristics in ESCC, patients were divided into the low PRAF2 expression group (*n* = 41, fold-change ≤ average ratio) and the high PRAF2 expression group (*n* = 36, fold-change > average ratio) (Fig. 1c). Statistical analysis revealed that PRAF2 was closely correlated with differentiation grade (*P* = 0.049) and TNM stage (*P* = 0.009). However, PRAF2 expression was not related to other clinical characteristics (all *P* > 0.05) (Table 1). Moreover, overexpression of PRAF2 was associated with lower differentiation grade and higher TNM stage (Fig. 1d, e).

**Table 1:** Clinical characteristics associated with PRAF2 mRNA expression

Characteristics	All patients	PRAF2 level			Overall survival	
		Low	High	*P* value	mOS (95% CI)	*P* value
Patients, No.	77	41	36			
Age, y						
≤ 60	34	16	18	0.366	28.0 (16.7–39.3)	0.144
> 60	43	25	18		26.0 (17.0–35.0)	
Gender						
Males	59	34	25	0.166	28.0 (22.2–33.8)	0.234
Females	18	7	11		22.0 (20.2–23.8)	
Location						
Up	10	3	7	0.192	29.0 (13.4–38.6)	0.624
Middle	45	25	20		26.8 (19.5–34.0)	
Low	22	13	9		30.0 (14.1–45.9)	
G stage						
G1	21	16	5	0.049*	36.0 (20.1–33.9)	0.009*
G2	45	20	25		22.0 (11.1–32.9)	
G3	11	5	6		17.3 (12.3–22.4)	
TNM stage						
I	4	4	0	0.009*	25	0.010*
II	52	31	21		30.0 (23.7–36.3)	
III	21	6	15		20.0 (15.2–24.8)	
PRAF2 level						
Low					33.0 (24.2–41.8)	< 0.001*
High					20.0 (16.3–23.6)	

*mOS* median overall survival, *y* years, *G* differentiation grade. *P < 0.05 indicates significant correlation

### Correlation between PRAF2 expression and overall survival in ESCC patients

**Fig. 2:**
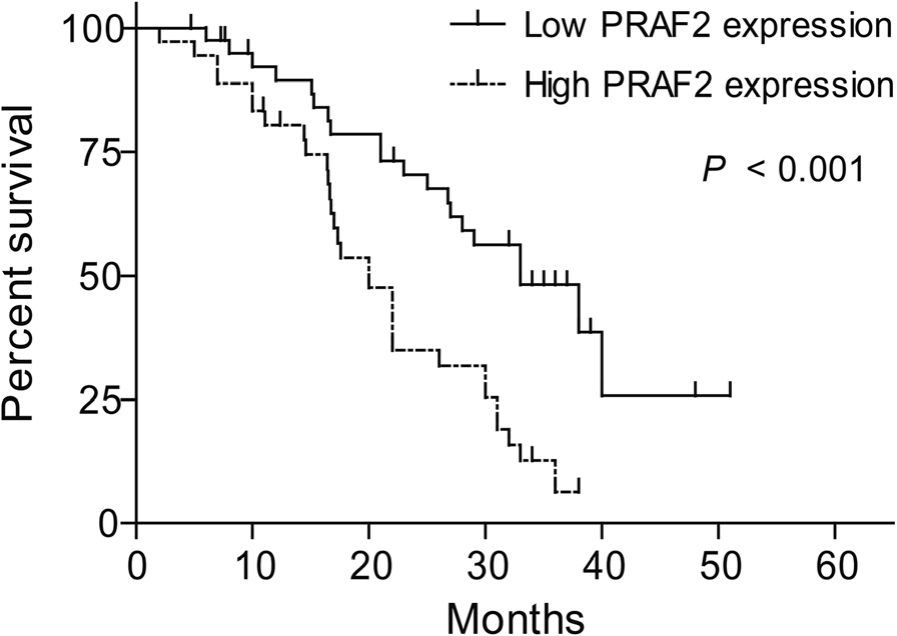
PRAF2 expression correlates with ESCC patient survival prognosis

Survival analysis indicated that ESCC patients with high PRAF2 mRNA expression had a decreased overall survival relative to those with low PRAF2 expression (median OS, 20.0 vs. 33.0 months, *P* < 0.001, Fig. 2). Also, we found that low differentiation (*P* = 0.009) and high stage (*P* = 0.010) were associated with the outcome of poor survival (Table 1). Then, multivariate analysis revealed that high PRAF2 mRNA expression (hazard ratio 2.05, 95% CI 1.10–3.85, *P* = 0.025) served as independent poor prognostic biomarker associated with decreased overall survival in patients with ESCC (Table 2).

**Table 2:** Multivariate cox regression analysis of clinical characteristics and PRAF2 expression associated with survival

Characteristics	HR	95% CI	*P* value
G stage (G3 vs. G2 or G2 vs. G1)	0.71	0.43–1.15	0.162
TNM stage (III vs. II or II vs. I)	1.53	0.85–2.76	0.153
PRAF2 (high vs. low)	2.05	1.10–3.85	0.025*

*G* differentiation grade, *HR* hazard ratios, *CI* confidence interval. *P < 0.05 indicates significant correlation

### Effect of PRAF2 on the ESCC cell proliferation in vitro

**Fig. 3:**
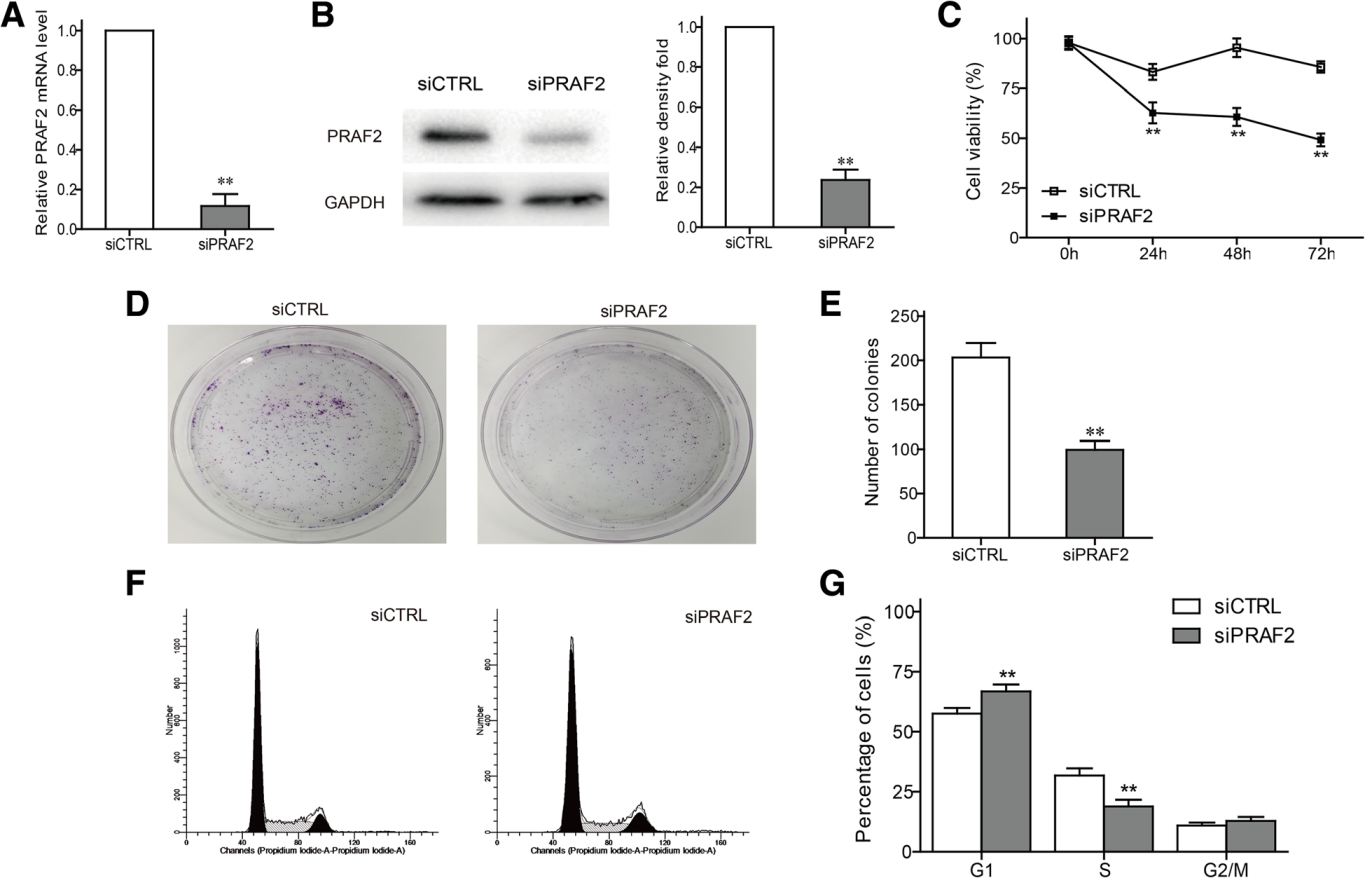
Effect of PRAF2 on proliferation and cell cycle in ESCC cells. **a** Relative PRAF2 mRNA expression was determined using qPCR. **b** PRAF2 protein was detected by western blot in TE1 cells with PRAF2 silencing. The relative density of PRAF2 was quantified and normalized to GAPDH. **c** CCK-8 assay was performed to determine the ability of cell proliferation. **c** Colony formation assay was performed to determine the clonogenic capacity of TE1 cells. **d** The bar chart represented the number of colonies. **e** The cell cycle distribution was determined using flow cytometry. **f** The histogram of quantitative analysis in different mitotic phases. Values represented the mean ± SD data in triplicate. **P < 0.01

To further explore the biological effect of PRAF2 in ESCC, we employed siRNA to silence PRAF2 expression in TE1 cells. Efficiency of silencing was estimated by qPCR and western blot, and PRAF2 siRNA was able to effectively decrease PRAF2 mRNA and protein expression (Fig. 3a, b). CCK-8 assay indicated knockdown of PRAF2 time-dependently inhibited the viability of TE1 cells (Fig. 3c). Similarly, PRAF2 silencing significantly inhibited the colony formation (Fig. 3d, e). Finally, the effect of PRAF2 on cell cycle progression was assessed by flow cytometry analysis. Knockdown of PRAF2 caused a G1 phase arrest in TE1 cells, and the cell number in S phase decreased (Fig. 3f, g).

### Effect of PRAF2 on the invasion potential of ESCC cells in vitro

**Fig. 4:**
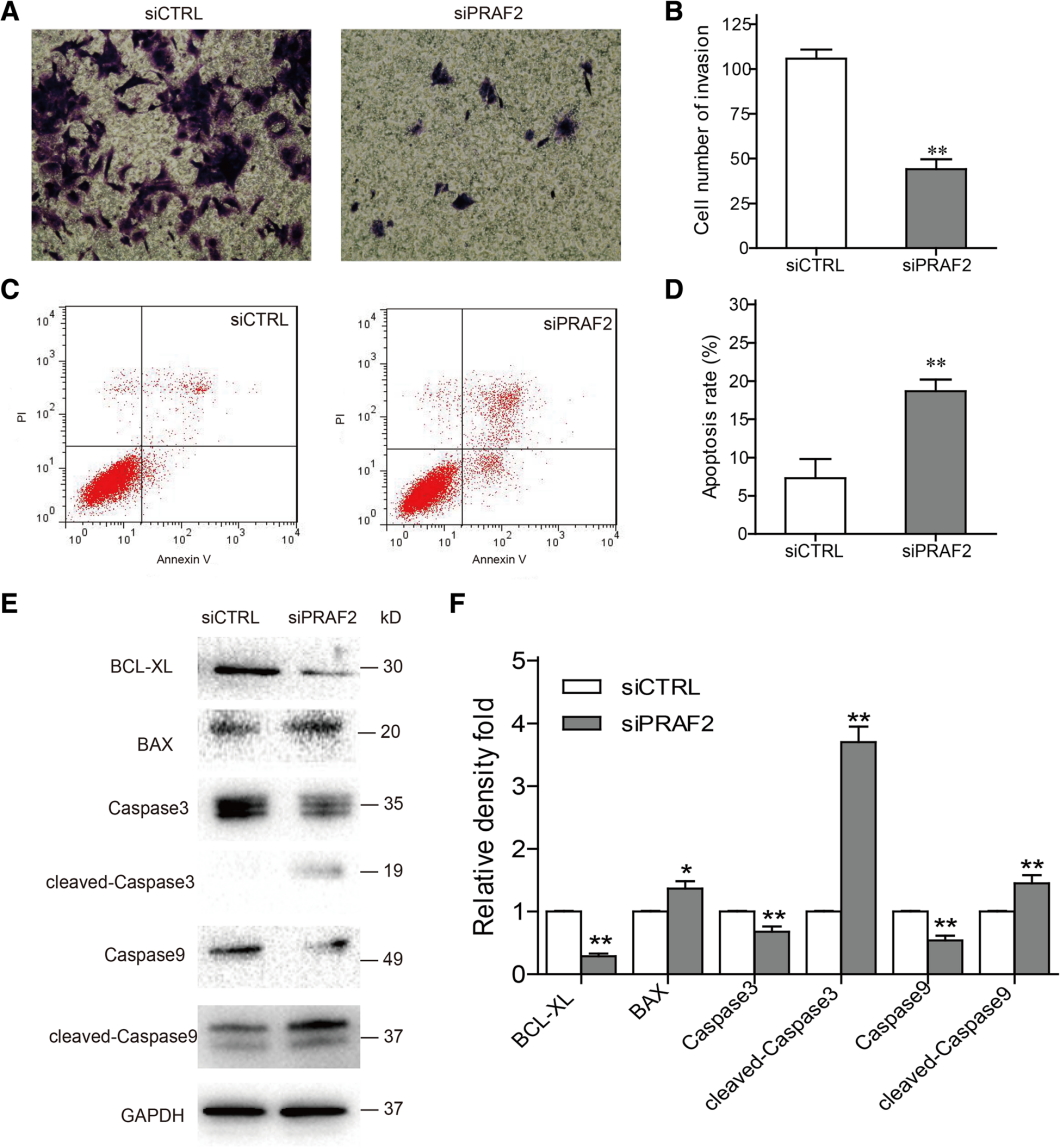
Impact of PRAF2 expression on ESCC cell invasion and apoptosis. **a** The effect of PRAF2 on cell invasion was determined by transwell assay, using cells with PRAF2 silencing. **b** The number of cells that migrated through the membranes was calculated and depicted in the bar chart. **c** Flow cytometry assay was performed to analyze apoptosis of TE1 cells with PRAF2 knockdown. **d** The bar chart represented the percentage of apoptosis. **e** The protein levels of apoptosis regulators were examined by western blot. **f** The relative densities of proteins were quantified and normalized to GAPDH. Values represented the mean ± SD data in triplicate. *P < 0.05 and **P < 0.01

To evaluate the effect of PRAF2 on invasion potential of TE1 cells, transwell assay was implemented. Our study showed that depletion of PRAF2 exhibited a remarkably decrease of invasion ability in TE1 cells (Fig. 4a, b). The result was a powerful indication that PRAF2 might participate in the process of metastasis of ESCC cells.

### Knockdown of PRAF2 induces apoptosis of ESCC cells in vitro

The function role of PRAF2 in cell apoptosis was assessed by flow cytometry analysis. Results demonstrated that PRAF2 silencing cause a remarkable induction of apoptosis in TE1 cells (Fig. 4c, d). To further explain the mechanism of apoptosis, several cell apoptosis-related proteins were examined by western blot. We discovered that anti-apoptotic protein of BCL-XL was downregulated, and pro-apoptotic proteins of BAX and cleaved-caspases (caspase3 and caspase9) were upregulated in TE1 cells after transfection of PRAF2 siRNA (Fig. 4e, f). PRAF2 might affect certain regulator’s expression to affect ESCC cell apoptosis.

## Discussion

ESCC is the most common histopathological subtype of esophageal cancer, and it has a high mortality rate largely due to late diagnosis [2, 5]. Therefore, there is a compelling need to screen for new early biomarkers and therapeutic targets. Here, for the first time, we demonstrated the prognostic significance and the biological action of PRAF2 in ESCC.

PRAF2 mRNA expression was remarkable increased in ESCC samples compared with associated non-tumor tissues. The finding was in agreement with the results reported in several human cancers [18–20]. Further, the prognostic value of PRAF2 was investigated and PRAF2 was recognized as a novel independent prognostic marker for poor survival in ESCC patients. Similar to our results, Wang et al. [20] discovered that PRAF2 overexpression was strongly associated with unfavorable prognosis in hepatocellular carcinoma. These results indicate that PRAF2 may be a good prognostic marker for patients with ESCC.

As we known, a critical mechanism underlying tumorigenesis is the disequilibrium in cell survival and death [24]. PRAF2 has been reported to be involved in the occurrence and progression of several solid cancers [18–20]. In the present study, PRAF2 silencing impaired ESCC cell proliferation and cell cycle. The result was consistent with studies in hepatocellular carcinoma and neuroblastoma [19, 20]. Though the function role of PRAF2 on cell apoptosis was not observed in tumors mentioned above, we found that knockdown of PRAF2 induced apoptosis of ESCC cells, and influenced the expression of apoptotic biomarkers which include BCL-XL, BAX, caspase3 and caspase9. These results indicated that PRAF2 silencing impaired ESCC cell growth via mitotic arrest and apoptosis. Further studies should be performed to clarify how PRAF2 involved in these functional roles.

Aggressive characteristic lead to tumor metastasis and then cause the poor prognosis. Our study has confirmed that high PRAF2 expression was associated with lower tumor differentiation grade and later TNM stage in ESCC. The results might suggest that PRAF2 was essential for ESCC progression. Also, PRAF2 silencing decreased the invasiveness of ESCC cells. Previous study shows PRAF2 interacts with the CCR5 and thereby contributes to cancer cell migration [8, 25], which may partially explain the mechanism of metastasis. Unlike the PRAF2, PRAF1, another member of PRA family, is known as a potential inhibitory factor of tumor cell invasion by regulating the MAPK and integrin αvβ3 pathways [26, 27]. Therefore, we speculated whether PRAF2 could function in reverse to regulate these pathways.

In summary, PRAF2 was an independent prognostic marker for poor survival in patients with ESCC. Further, we demonstrated that PRAF2 is a novel oncogene that facilitates cell growth and invasion, and inhibits apoptosis in ESCC cells.

## Conclusions

In present study, our data demonstrated that PRAF2 played a vital role in progression of ESCC and suggested PRAF2 might be a potential prognostic factor and treatment target for ESCC.

## Data Availability

The datasets used and/or analyzed during the current study are available from the corresponding author on reasonable request.

## Abbreviations

CI: Confidence interval
ESCC: Esophageal squamous cell carcinoma
HR: Hazard ratio
OS: Overall survival
PRAF2: Prenylated Rab acceptor 1 domain family member 2
